# A Rare Case of Subacute Sclerosing Panencephalitis in an Immunized Patient

**DOI:** 10.7759/cureus.63258

**Published:** 2024-06-26

**Authors:** Ruhi Shaligram, Balakrushna P Garud, Renuka S Jadhav, Shiji Chalipat, Shailaja Mane

**Affiliations:** 1 Pediatrics, Dr. D. Y. Patil Medical College, Hospital & Research Centre, Dr. D. Y. Patil Vidyapeeth (Deemed to be University), Pune, IND

**Keywords:** subacute sclerosing panencephalitis, encephalitis, mmr vaccine, epidemiology, measles vaccine

## Abstract

This study presents a case of subacute sclerosing panencephalitis (SSPE), a rare neurologic disorder characterized by brain inflammation, typically triggered by measles virus reactivation or an abnormal immune response to it.

This case involves a five-year-old male child with persistent fever, declining motor function, excessive sleepiness, and myoclonic jerks. MRI indicated potential ischemic changes or encephalitis, while electroencephalography showed SSPE-consistent patterns. Further investigations confirmed SSPE, with elevated IgG levels in serum and cerebrospinal fluid (CSF) and positive measles IgG antibodies in CSF. Treatment included isoprinosine, lamivudine, and intrathecal interferon-alpha for symptom management and disease progression. Despite atypical SSPE features, subclinical measles infection was considered a probable cause. The patient showed partial improvement post-treatment and was discharged for follow-up.

By reporting this case, we would like to emphasize clinical judgment, early detection of the symptoms, and lateral thinking to diagnose fatal conditions such as post-measles SSPE, even in fully immunized patients.

## Introduction

Subacute sclerosing panencephalitis (SSPE) is a rare and progressive neurological condition that arises from prolonged infection with a mutated form of the measles virus [1]. Typically, symptoms of SSPE emerge several years after the initial measles infection, characterized initially by intellectual decline and behavioral changes [2,3]. As the disease progresses, patients may experience generalized convulsions, coma, and dementia that can result in death usually within three years of symptom onset [1]. While rare, cases of prolonged spontaneous remission have been observed.

Diagnosis of SSPE is confirmed through a standardized clinical course along with specific indicators, including the detection of measles antibodies in the cerebrospinal fluid (CSF), electroencephalography (EEG), or histological findings in brain biopsy or postmortem examination [4]. These criteria help distinguish SSPE from other neurological conditions and aid in its accurate diagnosis and management.

SSPE disproportionately affects populations in poor and resource-constrained countries such as India. According to the World Health Organization, approximately 4-11 cases per 100,000 cases of measles are associated with SSPE [1]. Notably, individuals who contract measles at a very early age face a significant risk of developing SSPE. Additionally, the risk of SSPE following measles vaccination is substantially lower, with an estimated 0.7 cases per 1 million vaccine recipients over a six-year period [5]. These findings underscore the importance of measles vaccination in reducing the incidence of SSPE, particularly in regions with limited healthcare resources.

## Case presentation

A five-year-old male child presented with a 15-day history of high-grade fever, intermittently relieved by medications, accompanied by progressive neurological symptoms. He experienced difficulty walking and sitting without support, along with a progressive loss of balance. Additionally, the patient reported excessive sleepiness over the past 10 days. Despite being fully immunized, including receiving the measles vaccine, he had no prior history of measles infection.

Upon examination, the child appeared drowsy with a Glasgow Coma Scale score of 9, displaying myoclonic jerks in both upper and lower limbs, along with atonia and intentional tremors. Meningeal signs were absent. Higher function changes such as non-specific behavioral disturbances and increased restlessness were observed. Cranial nerve examination yielded normal results. Basic deep tendon reflexes were brisk and extensor plantar responses were extensor. In addition, frequent myoclonic drops were observed.

**Figure 1:**
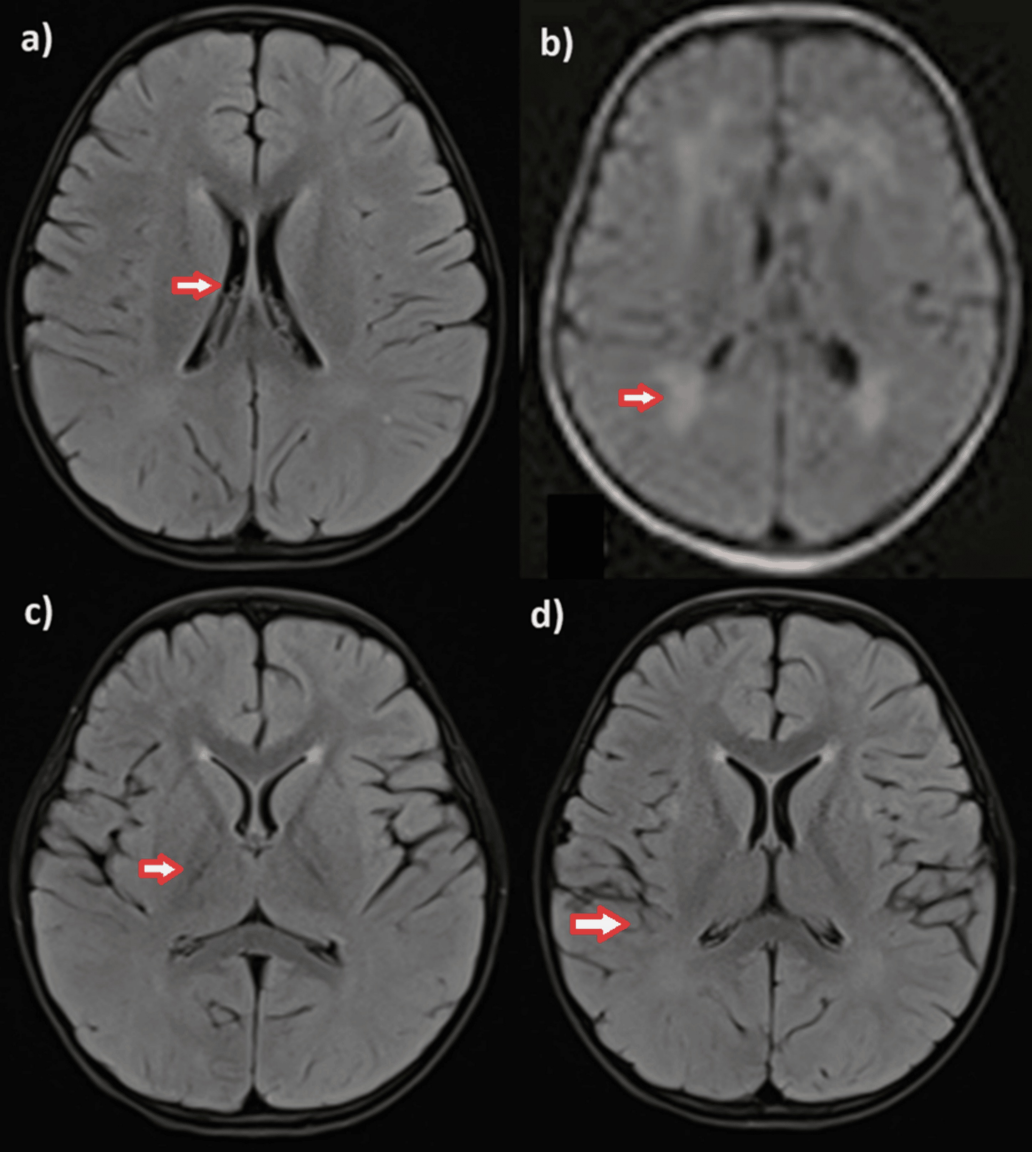
MRI images fluid-attenuated inversion recovery sequence. (a) Mild enlargement of lateral ventricles. (b) Hyperintensity in bilateral periventricular area. (c) subtle hyperintensity in the putamen area. (d) subcortical area involvement.

MRI findings revealed small hyperintensities in bilateral periventricular areas, suggestive of possible ischemic changes or encephalitis (Figure 1). The CSF analysis revealed elevated levels of total IgG (7.2) and positive measles IgG antibodies (4.98), indicative of SSPE (Table 1). Other CSF parameters, such as cell count, neutrophil/lymphocyte ratio, protein, glucose, adenosine deaminase, and lactate levels, were within normal ranges. The virology panel results were negative.

**Table 1:** CSF panel. CSF = cerebrospinal fluid; IgG = immunoglobulin G

Parameter	Result	Normal range
Serum total IgG	1,786	700–1,600
CSF total IgG	7.2	0–3.4
CSF measles IgG antibodies (+ve)	4.98	<1.5
Measles virus IgG (+ve)	>300	<300

Additional investigations did not reveal any abnormalities (Table 2). These findings, combined with the CSF results, confirmed the diagnosis of SSPE in the patient.

**Table 2:** Blood investigations. TLC = total leucocyte count; CRP = C-reactive protein; IgG = immunoglobulin G; N/L = neutrophil/lymphocyte; PCV = packed cell volume

Parameter	Value
Hemoglobin	13.5 g/dL
TLC	12,300/µL
Ca^2+^	10.3 mg/dL
CRP	3.12 mg/dL
Dengue	Negative
COVID-19 IgG	Negative
Platelets	4.44 lakhs
N/L	54/36
PCV	40.8%
Na	135 mEq/L
K	4.55 mEq/L
Cl	98 mEq/L
Blood culture	No microbial growth

**Figure 2:**
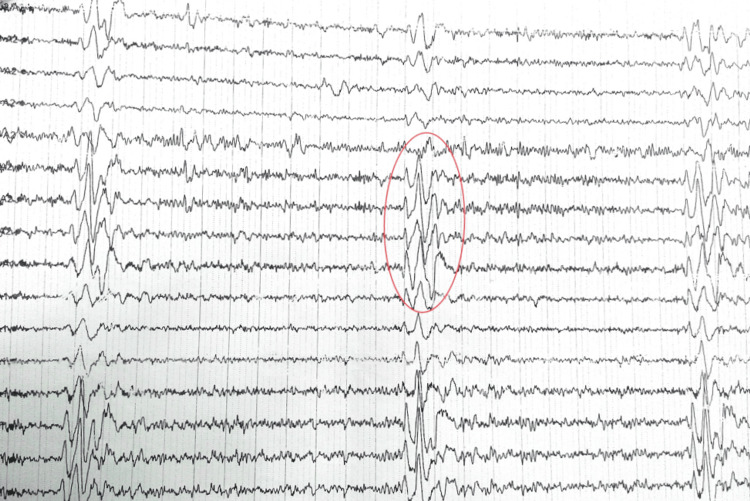
Electroencephalography recording. High-amplitude quasiperiodic slow wave complexes time locked with myoclonic jerks.

The EEG findings indicated high-amplitude quasiperiodic slow wave complexes synchronized with myoclonic jerks, suggestive of SSPE (Figure 2). This abnormal brain wave pattern was characteristic of SSPE and further supported the diagnosis in this case.

The patient was initiated on a treatment regimen consisting of isoprinosine, lamivudine, and intrathecal interferon-alpha. Isoprinosine acts by inhibiting protein synthesis, lamivudine works by inactivating viral RNA, and intrathecal interferon alpha enhances phagocytic and cytotoxic mechanisms. Symptomatic management was also provided to address specific symptoms. The primary goal of this treatment approach was to achieve partial improvement in symptoms or slow down disease progression.

Following the treatment course, the patient showed some improvement, and there were no further complications. Subsequently, the patient was discharged and scheduled for follow-up after one month to monitor progress and adjust treatment as necessary.

## Discussion

SSPE is a rare neurodegenerative condition resulting from persistent infection of the brain by a mutated form of the wild measles virus [6]. While the exact cause remains unclear, it is believed to stem from prolonged infection by the mutant virus, leading to the degeneration of white matter in the cerebral hemispheres and brainstem [7]. Despite high immunization rates in developed nations, SSPE remains a significant public health concern in developing countries [8]. Typically affecting children, SSPE often manifests years after a primary measles infection, with symptoms progressing through stages of personality changes, seizures, intellectual disabilities, rigidity, and, eventually, coma and death [1]. As the disease progresses, patients frequently develop pyramidal and extrapyramidal signs. They become spastic, quadriplegic, and myoclonus may disappear. In the later stages, breathing becomes noisy and irregular, and decerebrate and decorticate posture may develop. The progression of the disease can be graded depending on the clinical presentation, as shown in Table 3.

**Table 3:** Staging of subacute sclerosing panencephalitis.

Stages	Clinical progression
Stage 1	Behavioral changes and cognitive decline
Stage 2	Myoclonus and motor deterioration
Stage 3	Pyramidal and extrapyramidal manifestations, the disappearance of myoclonus, and disorientation in sensorium
Stage 4	A vegetative state

The criteria for the diagnosis of SSPE were proposed by Dyken [9], as presented in Table 4.

**Table 4:** Dyken’s modified criteria for diagnosing subacute sclerosing panencephalitis.

Major	
Clinical	Progressive, subacute mental deterioration with typical signs such as myoclonus
Measles antibodies	Raised titers in serum (>1:256) and/or cerebrospinal fluid (>1:4)
Minor	
Electroencephalography	Periodic, stereotyped, high-voltage discharges
Cerebrospinal fluid	Raised gamma-globulin or oligoclonal pattern
Brain biopsy	Suggestive of panencephalitis
Special	Molecular diagnostic test to identify the measles virus mut
Diagnostic requirement: two major plus one minor criterion are required, but if the features are atypical, then histopathological or molecular evidence may be required	

In our case, the patient presented with myoclonus and intellectual disabilities in stage 2 and progressed to stage 3, highlighting the insidious nature of the disease even in vaccinated individuals.

In one of the studies [10], successful measles vaccination programs protect the population against SSPE and have the potential to eliminate SSPE through the elimination of measles. Epidemiological and virological data suggest that measles vaccine does not cause SSPE. Another study [11] emphasized the role of vaccination in preventing progression into SSPE.

The case we are reporting makes us believe that despite effective coverage of measles immunization in our country, a fatal disease such as SSPE continues to affect fully vaccinated children. This case alerts the clinician to consider the possibility of SSPE even in younger and fully vaccinated children with cognitive decline and myoclonus.

One of the limitations in the treatment of SSPE is the difficulty in recognizing early manifestations of the disease when the inflammatory changes are still reversible.

The families of SSPE patients face significant physical, psychological, and financial burdens, necessitating extensive support systems to help them cope with these challenges. Ultimately, effective measles vaccination remains the primary preventive measure against this devastating neurological disorder, emphasizing the critical role of vaccination programs in safeguarding public health.

## Conclusions

Our case exhibited three atypical features of SSPE: an early age at onset, acute ataxia indicative of a rapid clinical course, and occurrence in a previously vaccinated child without a documented history of measles infection. We believe the possibility of subclinical measles infection overlooked by parents contributed to the development of SSPE. This highlights the complexity of SSPE diagnosis in vaccinated individuals and underscores the need for increased clinical suspicion and comprehensive evaluation in such cases.
